# Peer Review of “Large Language Models for Pediatric Differential Diagnoses in Rural Health Care: Multicenter Retrospective Cohort Study Comparing GPT-3 With Pediatrician Performance”

**DOI:** 10.2196/73264

**Published:** 2025-03-19

**Authors:** Daniela Saderi, Goktug Bender, Toba Olatoye, Arya Rahgozar, Uday Kumar Chalwadi, Eudora Nwanaforo, Paul Hassan Ilegbusi, Sylvester Sakilay, Mitchell Collier

**Affiliations:** 1 PREreview, Portland, OR, United States; 2 McGill University, Montreal, ON, Canada; 3 University of Ilorin, Ilorin, Nigeria; 4 University of Ottawa, Ottawa, ON, Canada; 5 LSUHS, Shreveport, LA, United States; 6 Federal University of Technology, Owerri, Nigeria; 7 Ondo State College of Health Technology, Akure, Nigeria

**Keywords:** natural language processing, NLP, machine learning, ML, artificial intelligence, language model, large language model, LLM, generative pretrained transformer, GPT, pediatrics

*This is the peer-review report for “Large Language Models for Pediatric Differential Diagnoses in Rural Health Care: Multicenter Retrospective Cohort Study Comparing GPT-3 With Pediatrician Performance.”*

This review is the result of a virtual collaborative live review organized and hosted by PREreview and JMIR Publications on October 25, 2024. The discussion was joined by 21 people: 2 facilitators, 1 member of the JMIR Publications team, and 18 live review participants, including 3 who agreed to be named here but did not contribute to writing this review: Nour Shaballout, Randa Salah Gomaa Mahmoud, and Samaila Jackson Yaga. The authors of this review have dedicated additional asynchronous time over the course of 2 weeks to help compose this final report using the notes from the live review. We thank all participants who contributed to the discussion and made it possible for us to provide feedback on this preprint.

## Summary

The study [1] seeks to determine how accurately and reliably a fine-tuned GPT-3 model can assist with differential diagnosis in pediatric cases within rural health care environments. Specifically, it examines whether the artificial intelligence (AI) model can match or approach the diagnostic accuracy of human physicians. By evaluating the model’s diagnostic performance, the research aims to explore AI’s potential to improve pediatric health care quality, reduce misdiagnosis, and support providers in underserved regions where accurate, timely diagnosis is critical for patient outcomes.

To address the research questions, the authors conducted a retrospective study using data from 500 pediatric cases from a multicenter rural pediatric health care organization in Central Louisiana, United States. The GPT-3 model was trained on 70% of the data, including symptoms and physician-provided differential diagnoses, and tested on the remaining 30%, achieving an accuracy of 87%, with sensitivity at 85% and specificity at 90%. These results were statistically comparable to human physicians, who had an accuracy of 91%. The findings suggest that AI can support clinical decision-making in pediatric care, especially in resource-constrained environments where access to specialists is limited.

The research addresses critical gaps in pediatric care by exploring AI’s potential to support clinical decision-making, particularly in resource-limited settings. It presents this with methodological details that enhance reproducibility and offer insights into AI applications in health care. The authors’ transparency about limitations reflects research integrity, establishing a strong base for future studies. Furthermore, the focus on integrating AI into clinical workflows shows an understanding of practical challenges and underscores opportunities for advancing health care delivery through technology. However, the study presents some notable weaknesses, including a lack of assessment of patient outcomes and insufficient clarity in its methodology, indicating areas for future research and improvement. Below, we list specific concerns and recommendations on how to address them.

## List of Major Concerns and Feedback

### Concerns With Techniques and Analyses

- Model choice: It is unclear why a specific generative AI model (ie, GPT-3, DaVinci version) was chosen for this study. Was the GPT-3 model (DaVinci version) selected due to its extensive use in medical AI research, or was it chosen to facilitate comparison with previous studies? A statement explaining the choice of the AI model would significantly improve the reader’s understanding of the study’s context and its relationship to previous research.
- Normality test: The study does not address whether data normality was assessed before statistical analysis. Determining the distribution of the data is key to selecting the appropriate statistical test to analyze such data. The Kolmogorov-Smirnov test could aid in understanding data distribution, specifically testing for normality. If the data is not found to meet normality criteria, nonparametric methods should be applied. Including a data normality assessment and explaining the choice of a particular statistical test would significantly strengthen the reliability of the study.
- Evaluation metrics: The study primarily uses specificity and sensitivity for evaluating large language model–generated responses, which may not capture the full quality of the outputs. Incorporating natural language processing metrics such as Recall-Oriented Understudy for Gisting Evaluation (ROUGE) and bilingual evaluation understudy (BLEU) can help assess the quality of generated responses more comprehensively. ROUGE measures the correspondence between the automatically generated response versus that of the human and what was expected. There are also issues associated with large language model generations of responses such as hallucination and the lack of attribution. Please specify or comment on how those and other issues were measured.
- Power analysis assumptions: The assumptions underlying the power analysis are unclear, particularly regarding how specific diagnoses affect this analysis. It is advised to elaborate on the power analysis methodology, including the rationale behind sample size choices and their implications for diagnosis variability.
- Sample size and generalizability: The sample size of 500 encounters may not adequately represent the broader pediatric population, particularly in diverse settings. Furthermore, using data from a single health care organization limits the applicability of findings to other settings. These limitations should be discussed, particularly how the validity of the results might change when it is tested with data from other health care centers. If possible, authors should mention and cite studies that reported on this effect. Additionally, future studies should consider expanding the sample size through multicenter collaborations or including data from patients with more diverse demographics to validate results across different health care environments thereby enhancing generalizability.

### Details for Reproducibility of the Study

- Software and tools documentation: The authors describe using both Python (with scikit-learn) and IBM SPSS Statistics, but it is unclear what the software’s sources are. Specifying sources for Python and scikit-learn (eg, “Python 3.8 [Python Software Foundation, Delaware, USA]”) and clarifying the respective roles of Python and SPSS in the analyses would enhance transparency and allow for the reproducibility of the study.
- Detailed group descriptions: The demographics, specifically age group cases, are underspecified, limiting the reader’s understanding of the study sample. Adding a table or descriptive text detailing subgroup demographics, including age and case counts would improve the study’s interpretability and allow readers to better contextualize findings.
- Cross-validation across organizations: The model’s reproducibility across various health care settings is not demonstrated. Evidence shows models often underperform with data from different sources. Including cross-organization validation and clearly acknowledging this limitation in the Discussion by citing relevant studies would enhance robustness. Furthermore, addressing this limitation in future work could pave the way for broader adoption and application of the model.
- Data and model specifics for replicability: The study would benefit from more thorough descriptions of dataset characteristics, fine-tuning model parameters, and preprocessing methods. For validation, consider adding multicenter dataset details. Adding this information would enable other researchers to replicate and build upon the study’s findings, thereby enhancing its scientific contribution.
- Diagnostic exclusion or inclusion clarification: The preprocessing section does not clarify if physician diagnostics were included or excluded, leading to potential confusion for readers and impacting reproducibility. It would be helpful to know whether physician diagnostics were included in training and why. Clarifying this aspect would help standardize study replication and improve the study’s transparency.

### Figures and Tables

- Figure 1 is mentioned but not included in the article, which affects comprehension of the study design and findings. Please include Figure 1 or provide an alternative reference to explain the content of the missing figure. Figures are helpful for readers to quickly grasp complex methodologies and findings.

### Ethics

- Data privacy: It is unclear whether a private or public version of GPT-3 was used, and if the latter, this raises potential Health Insurance Portability and Accountability Act (HIPAA) concerns. As was already pointed out above, it is recommended that the version of GPT-3 used is specified, with additional clarification regarding data privacy practices if a public model was used. The addition of HIPAA considerations will enhance readers’ confidence in the study’s privacy protocols.
- Discussion of diagnostic risk: The discussion would benefit from a deeper exploration of diagnostic risks associated with the use of AI in health care and clinical decision-making settings. One example is the potential of AI models to perpetuate and affirm existing human biases thereby further exacerbating health disparities (one relevant citation could be Mittermaier M, Raza MM, Kvedar JC. Bias in AI-based models for medical applications: challenges and mitigation strategies. *NPJ Digit Med.* Jun 14, 2023;6(1):113 [doi: 10.1038/s41746-023-00858-z] [Medline: 37311802]). The study also raises important social considerations, such as respecting human agency, particularly for vulnerable populations. Addressing parental concerns about deferring decision-making to AI is crucial, as is ensuring a socially attuned approach to building trust and understanding.
- Lack of clarity on potential implementation in rural health care settings: The study could be strengthened by detailing how the AI model might be implemented in rural health care settings, including the specific challenges involved. Key considerations include the need for sufficient infrastructure (eg, electricity, internet) and the necessity of training health care providers unfamiliar with AI tools. Additionally, discussing both the potential impact (eg, improved diagnostic efficiency) and limitations (eg, handling incomplete data or overreliance on AI) would provide a more comprehensive road map for deployment in rural environments.

## List of Minor Concerns and Feedback

- Data distribution gaps: No comparison of racial identity distribution between training and testing sets. Please consider adding a table or section on these demographic comparisons to ensure representation across subgroups.
- Data description and context: It would be helpful to know more information regarding how physicians were selected and their specific roles in the study.
- Departmental affiliations: Authors’ affiliations lack specific department details, which limits transparency. Include departmental affiliations for authors to increase transparency and traceability. Adding departmental affiliations will provide context on the authors’ expertise and institutional support.
- Funding transparency: The funding statement does not clearly specify whether the study was internally or externally funded. Explicitly state funding details, clarifying internal/external sources as applicable. Clear funding information will enhance transparency and address potential conflicts of interest.
- Approval number: While an ethical approval statement is present, it lacks the approval number, which is critical for ethical transparency. Please include the ethics approval number/code to ensure proper documentation and strengthen the study’s validity and trustworthiness.
- Inconsistent data collection dates between the abstract and data collection section (lines 19 and 82)
- Missing figure (line 104).
- Need for more descriptive statistics (mean, median, quartiles, SD).
- Data distribution: Lack of comparison for racial/Hispanic identity distribution between training and testing sets. There’s insufficient detail on age subgroup distribution.
- Clarification needed: The authors need to provide a deeper discussion of the power analysis methodology.
- The authors assessed that the distribution of age, gender, and chief complaints was similar between the training and testing sets. Suggest this to be cited to Table 5.
- Table 1: The abbreviations in the formula column should be identified in the table legend as “(FN: False Negative; FP: False Positive; TN: True Negative; TP: True Positive) (m)+1.”
- Please clarify why GPT-3.5 or GPT-4 (instead of GPT-3) was not used despite being available at the time of the study.
- Line 103 states physicians were instructed to generate differential diagnoses. I thought this was obtained retrospectively. Please clarify.
- Line 152: Table 4 should be corrected to Table 3.
- Line 154: Table 5 should be corrected to Table 4.
- Line 200: Typo “may limit the of the finding.”

## Concluding Remarks

We thank the authors of the preprint for posting their work openly for feedback. We also thank all participants of the live review call for their time and for engaging in the lively discussion that generated this review.

## Abbreviations

AI: artificial intelligence
BLEU: bilingual evaluation understudy
HIPAA: Health Insurance Portability and Accountability Act
ROUGE: Recall-Oriented Understudy for Gisting Evaluation
